# RpoE fine tunes expression of a subset of SsrB-regulated virulence factors in *Salmonella enterica *serovar Typhimurium

**DOI:** 10.1186/1471-2180-9-45

**Published:** 2009-02-26

**Authors:** Suzanne E Osborne, Brian K Coombes

**Affiliations:** 1 Michael G. DeGroote Institute for Infectious Disease Research, Department of Biochemistry and Biomedical Sciences, McMaster University, Hamilton, ON, Canada; 2 Laboratory for Foodborne Zoonoses, Public Health Agency of Canada, Guelph, ON, Canada

## Abstract

**Background:**

The survival of *Salmonella enterica *within the intracellular host niche requires highly co-ordinated expression of virulence effectors predominantly regulated by the SsrAB two-component regulatory system. *S. enterica *serovar Typhimurium mutants lacking the *ssrAB *genes are avirulent in mice, highlighting the importance of this regulatory system *in vivo*. Mutants lacking the gene encoding the alternative sigma factor σ^E ^(*rpoE*) are also highly attenuated for intracellular survival, pointing to a potential connection with the SsrAB regulatory system.

**Results:**

In this study we demonstrate that RpoE is involved in fine-tuning the expression of a subset of SsrB-regulated genes found in the *Salmonella *pathogenicity island-2 (SPI-2) genetic locus that encodes a horizontally acquired type III secretion system, and unlinked genes integrated into this regulon that are required for virulence in host animals.

**Conclusion:**

These data point to a potential connection between the virulence phenotype of strains lacking *ssrB *and *rpoE*, and highlight new transcriptional regulation that might be essential for appropriate temporal and spatial control of the virulence-associated type III secretion system during host infection.

## Background

*Salmonella enterica *are enteric pathogens that acquired a type III secretion system (T3SS) through horizontal gene transfer of a genomic island termed *Salmonella *Pathogenicity Island 2 (SPI-2) [1,2]. The SPI-2-encoded T3SS and its translocated effectors modify the intracellular host niche for *Salmonella *replication [3-5]. SPI-2 also has genes, *ssrA *and *ssrB*, which code for SsrAB, a two-component regulatory system needed for expression of the T3SS [6,7]. SsrB regulates the expression of SPI-2 encoded substrate effectors including *ssaB*, as well as several integrated virulence effectors such as *sseL *[8] and *srfN *[9] that are encoded elsewhere on the chromosome but that have integrated into the SsrB regulon. Mutants lacking *ssrAB *are unable to survive within macrophages and are avirulent in mice [1].

Alternative sigma factors coordinate gene expression in response to environmental cues sensed by the bacterium. Sigma factors have a specific recognition motif at the -35 and -10 positions and function to concentrate RNA polymerase at a subset of promoters [10]. One alternative sigma factor, RpoE (σ^E^) responds to envelope stress at the cell surface. Release of σ^E ^from its inner membrane anchored anti-sigma factor, RseA, leads to induction of genes required to maintain cell envelope integrity [11]. SsrB-regulated translocated effectors protect *S*. Typhimurium against host cell defences such as oxidative stress and antimicrobial peptides that perturb bacterial membrane integrity and provide a stimulus for σ^E ^release [4,12-15]. Although proficient at cellular invasion, *rpoE *or *ssrB *mutants are highly attenuated for intracellular survival in both cultured cells and animal hosts [16]. In addition, the expression of *rpoE *and *ssrB *is up-regulated within macrophages [17]. Links between RpoE and virulence gene expression is evident in other bacterial systems as well. Deletion of *rseA *in *Yersinia pseudotuberculosis *causes elevated virulence effector synthesis and secretion [18], establishing links between alternative sigma factors and virulence-specific regulators. Taken together, a connection between σ^E ^and SsrB is suggested from the available literature, however the role of σ^E ^in activating SsrB-regulated genes has not been studied.

We tested the hypothesis that RpoE is involved in expression of genes that use the SsrB response regulator for activation. By testing six promoters representing four classes of SsrB-regulated promoters ((i) two type III secretion structural operons in SPI-2, (ii) the effector operon in SPI-2, (iii) two effector genes unlinked with SPI-2, and (iv) an integrated virulence gene unlinked with SPI-2) we demonstrate that RpoE elicits an effect on a subset of SsrB-regulated genes. This effect was bidirectional depending on the promoter and was downstream of *ssrB *expression itself, since deletion of *rpoE *had no effect on SsrB levels in the mutant cells. These data help unite the virulence phenotypes of strains lacking SsrB and RpoE, and highlight new transcriptional regulation that might be essential for appropriate temporal and spatial control of the virulence-associated type III secretion system during host infection.

## Results

### Deletion of *rpoE *affects a subset of SsrB-regulated virulence genes

**Figure 1:**
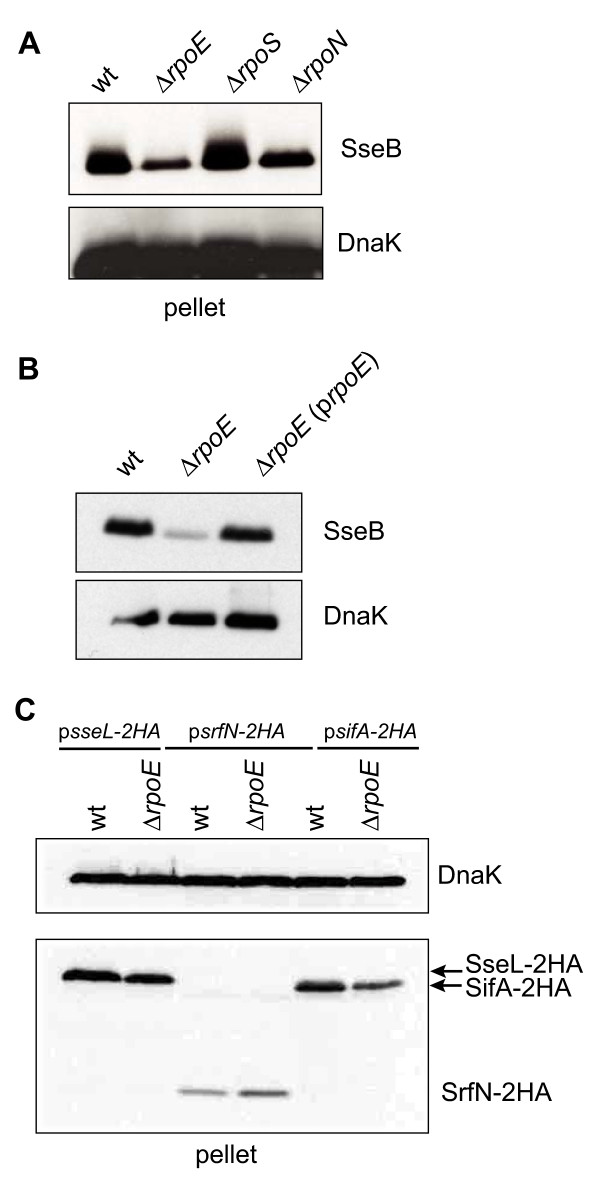
**Loss of *rpoE *changes the abundance of virulence factors in *Salmonella***. (A) wild type (wt), Δ*rpoE, ΔrpoS*, and *ΔrpoN S*. Typhimurium 14028s were grown for 6 hours under SsrB-inducing conditions. Lysates were probed by western blot for SseB, a component of the T3SS needle complex. (B) Western blot was performed as above and lysates probed for SseB in wild type (wt) *S*. Typhimurium SL1344, Δ*rpoE *and in Δ*rpoE *complemented with pWSK29 carrying full length *rpoE *with endogenous promoters. (C) Wild type *S*. Typhimurium SL1344 and Δ*rpoE *cells were immunoblotted as above and lysates probed for SseL-2HA, SrfN-2HA and SifA-2HA which were expressed from their endogenous promoters in pWSK29. Blots were probed for DnaK as a control. The experiment was performed three times with similar results.

*Salmonella *virulence gene expression is coordinated *in vivo *and may be regulated, in part, by alternative sigma factor(s) in order to quickly respond to the host environment. To date, no sigma factor has been identified as regulating SsrB-dependent virulence genes. To start, we first screened four alternative sigma factor mutants of *S*. Typhimurium (*rpoS, rpoN, rpoE, rpoH*) for their ability to express a key virulence gene, *sseB*, that requires SsrB for expression and whose gene product is essential for intracellular pathogenesis. For an *rpoH *deletion, this strain was only viable at temperatures below 30°C. Since SPI-2 gene activation is integrated into a thermosensing circuit [19] we were unable to test the role of σ^H ^in this study (data not shown). In this screen, *rpoS *deletion resulted in a slight increase in SseB levels (Figure 1A) indicating a role for RpoS in the repression of SPI-2. Both *rpoE *and *rpoN *deletions resulted in decreased SseB levels with a more pronounced effect in the *rpoE *deletion. Since we were predominantly interested in sigma factors that activate SPI-2 and which could be linked to the previous observation that *rpoE *mutants are highly attenuated *in vivo *we choose to focus on RpoE in the current study, which had the most influence on SseB levels in the cell.

An unmarked in-frame deletion of *rpoE *was then generated in *S*. Typhimurium strain SL1344 and we verified that this in-frame deletion had the same effect on SseB as the *rpoE*::*cat *mutant used previously (Figure 1B). A low-copy plasmid containing full-length *rpoE *and the three endogenous promoters that can drive its expression [20] was able to restore wild type levels of SseB to Δ*rpoE *cells (Figure 1B) demonstrating that the results were specific to the *rpoE *deletion. In these complementation experiments, attempts were made to examine the levels of SseB secreted into the culture supernatant [21], however consistent with previous observations [22,23] perturbations to the *rpoE *pathway increased cell lysis resulting in contamination of secreted fractions with cytosolic proteins which precluded accurate interpretation (data not shown).

In order to examine the effect of σ^E ^(*rpoE*) on the expression of a broad range of SsrB-regulated virulence genes, we tested whether or not the effect of *rpoE *deletion was specific to *sseB *or if it extended to other SsrB-regulated genes. To do this we examined the levels of SseL-2HA, SifA-2HA and SrfN-2HA expressed from their endogenous promoters under SPI-2 inducing conditions (Figure 1C). Consistent with the results for SseB, there was a decrease in SifA-2HA levels in Δ*rpoE *compared to wild type, although deletion of *rpoE *did not have an effect on SseL-2HA. Relative to its expression in wild type cells, the level of SrfN-2HA was reproducibly increased in the Δ*rpoE *cells, suggesting a role for σ^E ^in the repression of SrfN, although it is unlikely that this is through a direct mechanism.

### RpoE is involved in transcriptional activity of a subset of virulence genes

In order to confirm the effect of σ^E ^on the expression of a broad range of SsrB-regulated virulence genes, we used wild type and Δ*rpoE *cells and integrated into the chromosome individually six single-copy transcriptional fusions representing promoters for four classes of SsrB-dependent genes or operons ((i) type III secretion effector operon (*sseA*); (ii) structural operon I (*ssaB*); (iii) structural operon II (*ssaG*); (iv and v) effectors encoded outside of SPI-2 (*sseL *and *sifA*); and (vi) integrated virulence genes unlinked to SPI-2 (*srfN*) [9]. Transcriptional fusions to *lacZ *of the *sseA*, *ssaB*, *ssaG*, *sseL*, *sifA *and *srfN *promoters (mapped previously; [9,24] were integrated into the chromosome of wild type and Δ*rpoE *cells and then grown in the SsrB-activating medium LPM. The activity of each promoter was measured using a β-galactosidase assay during exponential growth. Although Δ*rpoE *was observed to have a slightly prolonged lag phase relative to wild type cells under these experimental conditions, at later time points the mutant grew similarly to wild type. To account for any differences in growth kinetics of the cultures, all data was normalized to the optical density at 600 nm of the culture, which permitted direct comparisons.

**Figure 2:**
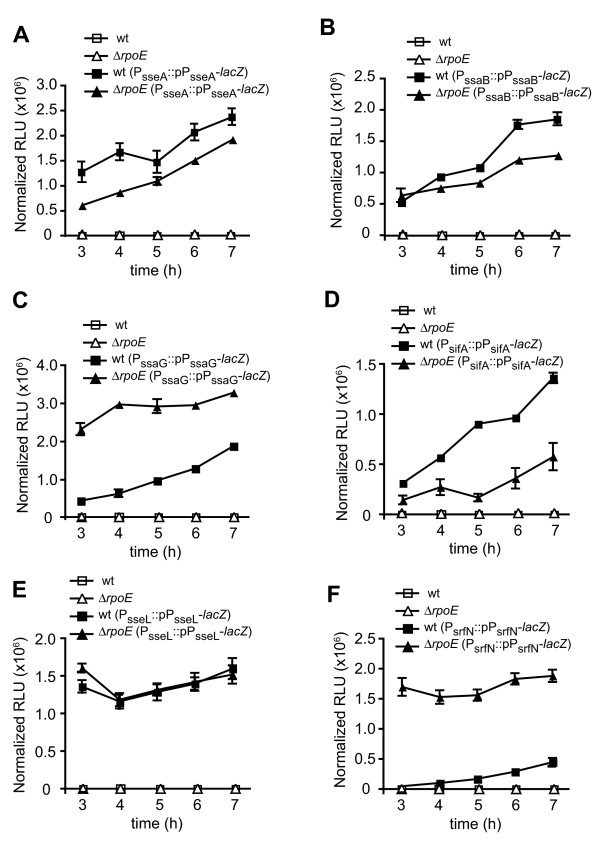
**The transcriptional activity of SsrB-regulated virulence genes is affected by an *rpoE *deletion**. Wild type and Δ*rpoE *cells carrying single-copy chromosomal transcriptional reporters of (A) P_sseA_::pP_sseA_-*lacZ*, (B) P_ssaB_::pP_ssaB_-*lacZ*, (C) P_ssaG_::pP_ssaG_-*lacZ*, (D) P_sifA_::pP_sifA_-*lacZ*, (E) P_sseL_::pP_sseL_-*lacZ *and (F) P_srfN_::pP_srfN_-*lacZ *were grown in LPM (pH 5.8). At the indicated time β-galactosidase activity was measured and expressed as relative light units (RLU) normalized to optical density of the culture. Wild type and Δ*rpoE *cells lacking the transcriptional reporters were used as controls in each experiment. Data are the means with standard error from triplicate determinations from three independent experiments.

In wild type cells, promoter activity from all the transcriptional fusions was high, as expected, because LPM medium is highly inducing for SsrB activity [21]. In contrast, promoter activity for *sseA*, *ssaB*, and *sifA *decreased in the *rpoE *mutant compared to wild type cells (Figure 2A, B and 2D), whereas promoter activity from the *ssaG *and *srfN *reporters was upregulated in the *rpoE *mutant (Figure 2C and 2F). β-galactosidase activity observed from the *sseL *reporter was unaltered in the *rpoE *deletion compared to that in wild type cells (Figure 2E). These data are consistent with the protein levels detected for these gene products. Together, these data indicate that σ^E ^can have a variable and bidirectional effect on SsrB-regulated virulence genes.

### The effect of RpoE on virulence genes is downstream of *ssrB *expression

**Figure 3:**
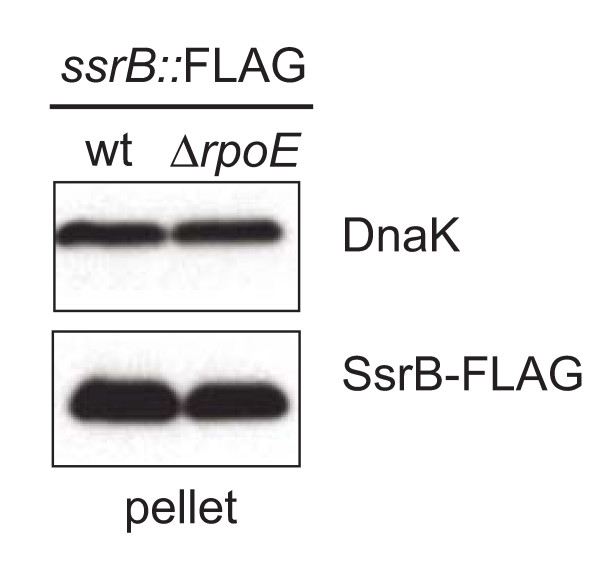
**The effect of RpoE on SsrB-regulated genes is downstream of *ssrAB *expression**. The *ssrB *gene in wild type and Δ*rpoE *cells was replaced with an *ssrB*-*FLAG *allele in its native location on the chromosome. Cells were grown under SsrB-activating conditions for six hours and lysates were probed by western blot to detect SsrB-FLAG and intracellular DnaK as a control. The data shown are representative of two experiments performed independently with identical results.

The variable effects of *rpoE *deletion on SsrB-regulated effectors suggested that RpoE might direct transcription downstream of *ssrB *expression. To test this, we replaced the *ssrB *gene in Δ*rpoE *and wild type cells with an *ssrB::FLAG *allele [19] and examined the levels of SsrB protein in the strains by western blot. There was no change in the levels of SsrB-FLAG between wild type and Δ*rpoE *cells (Figure 3), indicating that the effects of RpoE on the four classes of virulence gene promoters examined here was not mediated through changes to SsrB protein levels. Together these data establish a role for RpoE in the fine-tuning of virulence gene expression in *S*. Typhimurium.

## Discussion

In this work we found that the alternative sigma factor, σ^E^, is involved in fine tuning the expressing of a subset of SsrB-regulated virulence genes required for *Salmonella *pathogenesis. Although the effect of *rpoE *deletion on promoter activity in some cases was mild, we have previously shown that gene regulators providing only modest transcriptional input have a profound influence on bacterial fitness in a host animal [25]. In cases where the regulator is deleted, the loss of genetic fine-tuning causes incongruous changes in the timing and magnitude of virulence gene expression, leading to fitness loss and strong attenuation. We predict that RpoE confers a similar fine-tuning effect on *Salmonella *virulence gene expression that is required for optimal within-host fitness during infection.

**Figure 4:**
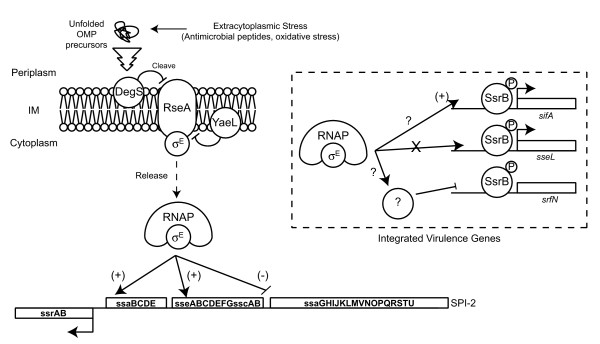
**Model for σ^E^-dependent regulation of the SsrB regulon**. Membrane-targeting host defences including reactive oxygen stress and antimicrobial peptides cause an accumulation of unfolded outer membrane proteins (OMPs) and stimulate the cleavage of the anti-sigma factor RseA consequently releasing σ^E ^into the cytoplasm where it directs RNA polymerase to a subset of SPI-2 promoters. RpoE can positively or negatively regulate SsrB-regulated genes including integrated virulence genes unlinked with SPI-2 but has no effect on some effector genes such as *sseL*. This regulatory pathway may have evolved to coordinate virulence gene expression with host infection by responding to host-specific defence pathways that perturb the bacterial outer membrane.

When we examined the -10 and -35 positions of the promoters studied here relative to the transcriptional start sites identified previously [24], these promoters did not appear to contain σ^E ^consensus sequences. Instead they appeared to have consensus sites for σ^70^. Although a bioinformatics screen identified σ^E ^consensus sequences upstream of the SPI-2 genes *ssaU*, *ssaJ*, *sscA *and *ssaC *[26], these genes were not tested for σ^E^-dependence in the present study because the identified consensus sites are in coding sequence within operons, and as a result may not be directly relevant. Due to the high degree of conservation in σ factor binding sequences, σ^E ^may not be directly regulating SsrB-dependent promoters. The lack of a canonical σ^E ^sequence at these promoters suggests that another regulatory gene may be epistatic to σ^E ^or that these promoters encode functional, but non-canonical σ^E^-binding sites due to their horizontal acquisition and gradual integration into the σ^E ^regulatory network. This integration may help *Salmonella *coordinate expression of the virulence-associated T3SS in response to host factors that compromise bacterial membrane integrity (Figure 4). This mechanism would activate a restorative σ^E ^pathway, which is consistent with the enhanced susceptibility of *rpoE *mutants to oxidative stress and antimicrobial peptides [13,15,16], both of which perturb membrane integrity *in vivo*. Although there is no evidence that σ^E ^can directly repress transcription, the negative effect on two promoters observed here might be due to an intermediate RpoE-regulated repressor or compensatory effect where loss of *rpoE *increases the relative abundance of another sigma factor that can directly activate the *ssaG *and *srfN *promoters. Future work will be required to resolve these possibilities.

Our results indicate that *rpoE *deletion has no effect on SsrB levels under SPI-2 inducing conditions suggesting that the σ^E ^pathway regulates effector expression downstream of *ssrAB *transcription. Unlinking *ssrAB *transcription from the σ^E ^regulon would be advantageous to the cell to prevent commitment to a virulence gene expression program in response to envelope stress not associated with infection. The results from this study demonstrate that σ^E ^has the ability to affect expression of SsrB-regulated virulence genes and offers potential insight into the virulence attenuation of *rpoE *mutants. Although when considered individually, each promoter was modestly affected by deletion of *rpoE*, the cumulative effects of mild rewired inputs on multiple virulence promoters has been shown to severely compromise in-host fitness and virulence ability [25].

## Conclusion

Based on these and other data [4,12-15], the genetic interaction between σ^E ^and a subset of SsrB-regulated genes may serve to coordinate the spatial and temporal activation of virulence genes in a host setting, likely in response to membrane damage resulting from oxidative anti-microbial systems and membrane-targeted host defence peptides.

## Methods

### Strains and Growth Conditions

Bacteria were propagated at 37°C with aeration in Luria-Bertani (LB) broth. *S*. *enterica *serovar Typhimurium (*S*. Typhimurium) strain 14028s with inactivating mutations in *rpoE*, *rpoS*, *rpoN *and *rpoH *were provided by Ferric Fang (University of Washington, Seattle, WA) [27]. Δ*rpoH *was grown at 30°C and Δ*rpoN *was supplemented with 2 mM L-glutamine. An unmarked, in-frame deletion of *rpoE *was made in *S*. Typhimurium strain SL1344 by λ Red recombination [28] using primers BKC187 and BKC188. Mutants were screened for loss of *rpoE *using primers BKC193 and BKC194. To generate an *ssrB::FLAG *allele in Δ*rpoE*, the *ssrB::FLAG *allele from wild type SL1344 [19] was transduced into Δ*rpoE *by P22-mediated transduction. All plasmids and strains used in this work are described in Table 1. Primer sequences for mutant and plasmid construction are listed in Table 2.

**Table 1:** Strains and plasmids used in this study

Strain or plasmid	Genotype or description ^a^	Reference
***Plasmids***		
		
pKD46	repA101ts, *ori*R101, *araC*-P_araB_-*gam-bet-exo, bla*	[28]
pCP20	FLP^+^, λ cl857^+ ^λ p_R _Rep^ts^, Amp^R^, Cm^R^	[28]
pKD3	pANTSy derivative, *FRT*-flanked *cat *from pSC140, Cm^R^	[28]
p*rpoE*	*rpoE *with endogenous promoters in pWSK29, Amp^R^	This work
p*srfN-2HA*	*srfN *with endogenous promoter and C-term. tandem HA fusion in pWSK129, Kan^R^	Our collection
p*sseL-2HA*	*sseL *with endogenous promoter and C-term. tandem HA fusion in pWSK129, Kan^R^	[8]
p*sseL-2HA*	*sifA *with endogenous promoter and internal tandem HA fusion in pACYC184	[37]
pIVET5n	*tnpR-lacZ*, *sacB*, R6K ori, *bla*, Amp^R^	[29]
pP_sseA_-*lacZ*	*sseA *promoter fused to *tnpR-lacZ *in pIVET5n, Amp^R^	[29]
pP_sseL_-*lacZ*	*sseA *promoter fused to *tnpR-lacZ *in pIVET5n, Amp^R^	This work
pP_srfN_-*lacZ*	*srfN *promoter fused to *tnpR-lacZ *in pIVET5n, Amp^R^	This work
pP_sifA_-*lacZ*	*sifA *promoter fused to *tnpR-lacZ *in pIVET5n, Amp^R^	This work
pP_ssaB_-*lacZ*	*ssaB *promoter fused to *tnpR-lacZ *in pIVET5n, Amp^R^	This work
pP_ssaG_-*lacZ*	*ssaG *promoter fused to *tnpR-lacZ *in pIVET5n, Amp^R^	This work
***Strains***		
SL1344	wild type *S. enterica *sv. Typhimurium, Sm^R^	[38]
14028s	wild type *S. enterica *sv. Typhimurium	[27]
14028s *rpoE*::*cat*	*rpoE *deletion, Cm^R^	[27]
140282 *rpoS*::Tn10dCm	*rpoS *deletion, Cm^R^	[27]
14028s *rpoN*::Ap	*rpoN *deletion, Amp^R^	[27]
14028s *rpoH*::PCN	*rpoH *deletion, temperature sensitive, Amp^R^	[27]
SL1344 Δ*rpoE*	Unmarked, in-frame deletion of *rpoE*, Sm^R^	This work
SL1344 *ssrB::3FLAG*	*ssrB-FLAG *replacement allele on chromosome	This work
SL1344 P_sseA_::pP_sseA_-*lacZ*	Merodiploid containing integrated P_sseA_-*lacZ *reporter	[25]
SL1344 P_sseL_::pP_sseL_-*lacZ*	Merodiploid containing integrated P_sseL_-*lacZ *reporter	This work
SL1344 P_srfN_::pP_srfN_-*lacZ*	Merodiploid containing integrated P_srfN_-*lacZ *reporter	This work
SL1344 P_sifA_::pP_sifA_-*lacZ*	Merodiploid containing integrated P_sifA_-*lacZ *reporter	This work
SL1344 P_ssaB_::pP_ssaB_-*lacZ*	Merodiploid containing integrated P_ssaB_-*lacZ *reporter	This work
SL1344 P_ssaG_::pP_ssaG_-*lacZ*	Merodiploid containing integrated P_ssaG_-*lacZ *reporter	This work
Δ*rpoE *P_sseA_::pP_sseA_-*lacZ*	Merodiploid containing integrated P_sseA_-*lacZ *reporter	This work
Δ*rpoE *P_sseL_::pP_sseL_-*lacZ*	Merodiploid containing integrated P_sseL_-*lacZ *reporter	This work
Δ*rpoE *P_srfN_::pP_srfN_-*lacZ*	Merodiploid containing integrated P_srfN_-*lacZ *reporter	This work
Δ*rpoE *P_sifA_::pP_sifA_-*lacZ*	Merodiploid containing integrated P_sifA_-*lacZ *reporter	This work
Δ*rpoE *P_ssaB_::pP_ssaB_-*lacZ*	Merodiploid containing integrated P_ssaB_-*lacZ *reporter	This work
Δ*rpoE *P_ssaG_::pP_ssaG_-*lacZ*	Merodiploid containing integrated P_ssaG_-*lacZ *reporter	This work

^a^Sm, streptomycin; Amp, ampicillin; Cm, chloramphenicol; Kan, kanamycin

**Table 2:** Primers used in this study

Primer	Sequence^ab^
BKC183	GCTCGAGTTTACCGCGCCGGATAACATACCG
BKC184	CACCAATTGTTAGCTGAATGAAGCAACCGTTGCCAG
BKC185	CCTCGAGCGATTGCCGTCAAAGGTATTC
BKC186	CACCAATTGTTACTGATCACCGTTCTCTACGGCGCT
BKC187	**GGTTTGGGGAGACATTACCTCGGATGAGCGAGCAGTTA**GTGTAGGCTGGAGCTGCTTCG
BKC188	**CCTAATACCTTTTCCAGTATCCCGCTATCGTCAACG**CATATGAATATCCTCCTTA
BKC195	CAGCTCACTATCCAACGTTT
BKC196	TGCTTGCTCATAGTGCGGCTT
BKC205	CACCAATTGTTACTATAGTAATCGGCATATTAA
BKC206	CCGCTCGAGTATGGCGCTGATCGCCAC
SEO005	CCGCTCGAGGTGCCATCCTTTGCCGTTT
SEO006	CACCAATTGTTACATGAATCCCTCCTCAGACAT
SEO011	CCGCTCGAGATATGGAGAGTGGTAGAATAG
SEO012	CACCAATTGTTATAATTGTGCAATATCCATAA
SEO095	GCTCTAGATCAACGCCTGATAAGCGGTT
SEO096	ACGCGTCGACCCACTCTTTATTGCGATTCCA

^a^underlined nucleotides indicate restriction enzyme sites; Xho*I *(CTCGAG), Mfe*I *(CAATTG)

^b ^bolded nucleotides indicate complementarity to *rpoE*

### Plasmid Construction

A complementation construct for *rpoE *was generated that included the three endogenous promoters (rpoEp1, rpoEp2, rpoEp3) identified in *Salmonella *[20] plus the wild type *rpoE *gene. This construct was cloned into the low-copy plasmid pWSK29 using primers SEO095 and SEO096 as a *Sal*I and *Xba*I fragment. Constructs were verified by sequencing and transformed into *S*. Typhimurium SL1344 Δ*rpoE *and selected on LB agar with appropriate antibiotics. The promoters for *ssaB *(SEO005 and SEO006), *ssaG *(SEO011 and SEO012), *sifA *(SEO205 and SEO206), *sseL *(BKC185 and BKC186) and *srfN *(BKC183 and BKC184) were cloned into pIVET5n [29] to generate single-copy transcriptional fusions to *lacZ*. Reporters were transformed into *E. coli *SM10 λ*pir*, conjugated into SL1344 and merodiploid cells were selected on LB agar with appropriate antibiotics. Transcriptional fusions, including a previously constructed reporter for the *sseA *promoter [30], were integrated into the chromosome of wild type and Δ*rpoE *cells using homologous recombination. The promoters we chose use the SsrB response regulator for expression of the downstream gene or operon, and include both SPI-2-encoded and non-SPI-2-encoded virulence effectors representing structural apparatus genes and effector substrates of the type III secretion system [8,30-35]

### Chemiluminescent β-galactosidase Assay

Reporter strains were inoculated from an overnight culture into culture medium (LPM pH 5.8) that induces SsrB-dependent gene expression [21,36]. Cultures were propagated at 37°C for 7 hours and samples were taken hourly to measure β-galactosidase activity using a chemiluminescence assay described previously [25]. Data was expressed as relative light units (RLU) and was normalized to the optical density (OD_600 nm_) of the parent culture.

### Immunoblotting

To examine the protein levels of SseB, SseL, SrfN and SifA under SPI-2 inducing conditions, we used plasmids p*sifA*-2HA, p*sseL*-2HA and p*srfN*-2HA that were published previously (Table 1) [8,37]. These constructs express the given gene under the control of the endogenous promoter. Wild type and Δ*rpoE *cells were transformed with these plasmids and grown in LPM pH 5.8 at 37°C for 6 hours. Whole cell lysates were collected and analyzed by immunoblotting using anti-SseB (1:1000) [21] and anti-HA (1:1000, Covance) antibodies. Blots were probed for DnaK (1:3500, Stressgen) as a control.

## Authors' contributions

SEO designed and performed research, interpreted data and wrote the paper. BKC designed and interpreted research and wrote the paper. Both authors read and approved the final manuscript.
